# Electronic Wave-Packets in Integer Quantum Hall Edge Channels: Relaxation and Dissipative Effects

**DOI:** 10.3390/e23020138

**Published:** 2021-01-22

**Authors:** Giacomo Rebora, Dario Ferraro, Ramiro H. Rodriguez, François D. Parmentier, Patrice Roche, Maura Sassetti

**Affiliations:** 1 Dipartimento di Fisica, Università di Genova, Via Dodecaneso 33, 16146 Genova, Italy; rebora@fisica.unige.it (G.R.); sassetti@fisica.unige.it (M.S.); 2 SPIN-CNR, Via Dodecaneso 33, 16146 Genova, Italy; 3 Université Paris-Saclay, CEA, CNRS, SPEC, 91191 Gif-sur-Yvette, France; ramiro.rodriguez@college-de-france.fr (R.H.R.); Francois.Parmentier@cea.fr (F.D.P.); patrice.roche@cea.fr (P.R.)

**Keywords:** electron quantum optics, interaction effects, relaxation, dissipation

## Abstract

We theoretically investigate the evolution of the peak height of energy-resolved electronic wave-packets ballistically propagating along integer quantum Hall edge channels at filling factor equal to two. This is ultimately related to the elastic scattering amplitude for the fermionic excitations evaluated at different injection energies. We investigate this quantity assuming a short-range capacitive coupling between the edges. Moreover, we also phenomenologically take into account the possibility of energy dissipation towards additional degrees of freedom—both linear and quadratic—in the injection energy. Through a comparison with recent experimental data, we rule out the non-dissipative case as well as a quadratic dependence of the dissipation, indicating a linear energy loss rate as the best candidate for describing the behavior of the quasi-particle peak at short enough propagation lengths.

## 1. Introduction

The possibility to prepare, manipulate, and measure individual electronic wave-packets propagating along mesoscopic quantum channels opened the way to so-called electron quantum optics (EQO) [1,2,3,4,5,6,7]. In this framework, seminal quantum optics experiments, such as the Hanbury–Brown–Twiss [8] and the Hong–Ou–Mandel [9] interferometry, have been realized by using ballistic electrons [10,11,12,13,14].

In the integer quantum Hall (QH) regime, due to the external magnetic field, the electrons propagate chirally along edge states of a two-dimensional electron gas that is topologically protected [15]. This means that the propagation of the electrons occurs ballistically without backscattering and with a very long coherence length. This made it possible to reach an extremely high level of control of the design and evolution of electronic wave-packets and provided some hope about the possibility of using electronic excitations as flying qubits [7,16,17,18], i.e., as a controlled and trustful way to transport quantum information over relatively long distances [19]. However, the actual implementation of this idea in realistic solid-state devices is seriously undermined by the presence of interaction among the electrons in the system and with the external environment—effects with no parallel in the photonic case. The role of Coulomb interaction has been extensively discussed both in order to properly understand the experimental observations achieved for integer states at filling factor ν=2 [14,20,21,22,23,24] and to predict new features occurring in the strongly interacting fractional QH regime [25,26,27,28,29,30] or in more exotic low-dimensional systems [31,32,33,34,35]. Conversely, the role of energy dissipation towards external degrees of freedom of individual electrons is still largely unexplored. However, a deeper understanding of the mechanisms associated with the energy leakage and the consequent loss of information carried by the electronic wave-packets, which severely compromises the possibility of using them as qubits [36], is needed in order to implement new and effective experimental designs that are able to reduce these detrimental effects in the same spirit as what was proposed for interaction effects in Ref. [22]. This could allow the development of more refined and robust paths towards quantum computation by exploiting the topological nature of integer and fractional QH edge states [37].

The preliminary steps in the study of dissipative effects involved the investigation of the evolution of a non-equilibrium electronic distribution as a function of the interaction length [38,39], where experiments showed that the signature of important energy losses towards external degrees of freedom was only included effectively in the theoretical models [40,41,42]. Such dissipation effects are crucial in order to properly describe both the dynamics of integer QH states [43] and the evolution of the peak height of energy-resolved wave-packets injected into them [44]. Remarkably enough, the predicted functional form of the dissipation as a function of the energy is different in these two cases; namely, it is quadratic in the former case and linear in the latter. Therefore, a more detailed analysis is needed in order to clarify this apparent discrepancy and to improve our understanding of this topic. Moreover, the possible physical origin of such energy losses has recently been attributed to the formation of a compressible strip close to the incompressible edge states due to smooth confining potential [45] or to inelastic non-local Auger-like processes that redistribute energy between spatially separated parts of the sample [46]. However, this latter mechanism seems to be relevant at longer propagation lengths than the one considered in Ref. [44]. Therefore, the subject of the dominant microscopic dissipation processes at different propagation lengths is still an open issue.

The present paper aims to shed new light on this subject. Assuming a phenomenological approach, we start from a hydrodynamic model, where the two edge channels are capacitively coupled through a short-range interaction [47]. In addition, we consider three possible dissipation regimes: the non-dissipative case, which is used as a reference case, an ohmic dissipation linear in the injection energy of the electronic wave-packet, and a quadratic dissipation. We observe that the linear dependence provides the best fit for the experimental data of the evolution of the experimental peak height at small enough propagation lengths [44]. Conversely, at greater propagation lengths, a dissipation quadratic in the injection energy dominates [43]. This apparent discrepancy could be related to both different sample designs and the involvement of more mechanisms of dissipation [45,46].

The paper is organized as follows. In Section 2, we discuss the edge state at ν=2, where the two channels are capacitively coupled, in terms of a bosonic hydrodynamic model. Section 3 describes the edge–magnetoplasmon scattering matrix that connects the bosonic fields incoming into the interacting region with the outgoing ones. Here, we also include the effects of energy dissipation towards external degrees of freedom. In particular, we consider a non-dissipative case and dissipation with a linear and a quadratic dependence on the injection energy. The elastic scattering probabilities for the fermionic excitations in the various regimes are reported in Section 4, and a comparison with experimental data is given in Section 5. Section 6 is devoted to the conclusion, while we have included technical details of the derivation of the elastic scattering amplitude in Appendix A.

## 2. Model

We consider the two edge channels of a QH bar at filling factor ν=2, assuming a short-range (δ-like) capacitive coupling between them. Considering the conventional Wen hydrodynamical approach [47], for this system, one can write the Hamiltonian density (ℏ=1) [48]:

(1) $$H\left(x\right)=\frac{v_{1}}{4\pi }{\left(\partial_{x}\phi_{1}\left(x\right)\right)}^{2}+\frac{v_{2}}{4\pi }{\left(\partial_{x}\phi_{2}\left(x\right)\right)}^{2}+\frac{u}{2\pi }\partial_{x}\phi_{1}\left(x\right)\partial_{x}\phi_{2}\left(x\right)$$

where $\phi_{i}$ (i=1,2) are bosonic fields related to the *i*-th edge particle density through the condition

(2) $$\rho_{i}\left(x\right)=\frac{1}{2\pi }\partial_{x}\phi_{i}\left(x\right),$$

where $v_{i}$ are the bare propagation velocities of the bosonic modes along the two channels and *u* is the intensity of their coupling. Without loss of generality, in the following, we will assume that $v_{1}\geq v_{2}$.

The above Hamiltonian can be diagonalized by means of a rotation in the bosonic field space of the form

(3) $$R\left(\theta \right)=\left(\begin{array}{cc} \cos\theta & \sin\theta \\ -\sin\theta & \cos\theta \end{array}\right)$$

with a mixing angle satisfying

(4) $$\tan\left(2\theta \right)=\frac{2u}{v_{1}-v_{2}}.$$

The above condition naturally leads to two different regimes.

### 2.1. “Strongly Interacting” Regime

The condition typically indicated in the literature as “strongly interacting” is characterized by

(5) $$\theta =\frac{\pi }{4},$$

which can actually be achieved only in the symmetric case $v_{1}=v_{2}=v$. This limit is usually assumed as a working hypothesis in the majority of the theoretical papers [20,21,23,40,42,49,50,51] and leads to the eigenvelocities

(6) $$v_{\rho ,\sigma }=v\pm u,$$

where $v_{\rho }$ is associated with a charge eigenmode $\phi_{\rho }$, while $v_{\sigma }$ corresponds to a dipole eigenmode $\phi_{\sigma }$. It is worth noticing that the stability condition of the model, namely, the fact that both eigenvelocities need to be positive, leads to the further constraint v>u. This implies that the coupling between the channels cannot be arbitrary high, contradicting the conventional denomination.

Even if frequently used in order to fit experimental data [14,40,43], this approximation has been revealed to be too restrictive in some cases [52].

### 2.2. “Moderately Interacting” Regime

In order to relax the above constraints, one can assume, without loss of generality, $v_{2}=v$ and $v_{1}=\alpha v$, with α>1. Notice that for α=1, we recover the previous case. Under these conditions, the two eigenvelocities of the model become

(7) $$v_{\rho ,\sigma }=vf_{\rho ,\sigma }\left(\alpha ,\theta \right)$$

with

(8) $$f_{\rho ,\sigma }\left(\alpha ,\theta \right)=\left(\frac{\alpha +1}{2}\right)\pm \frac{1}{\cos\left(2\theta \right)}\left(\frac{\alpha -1}{2}\right).$$

**Figure 1:**
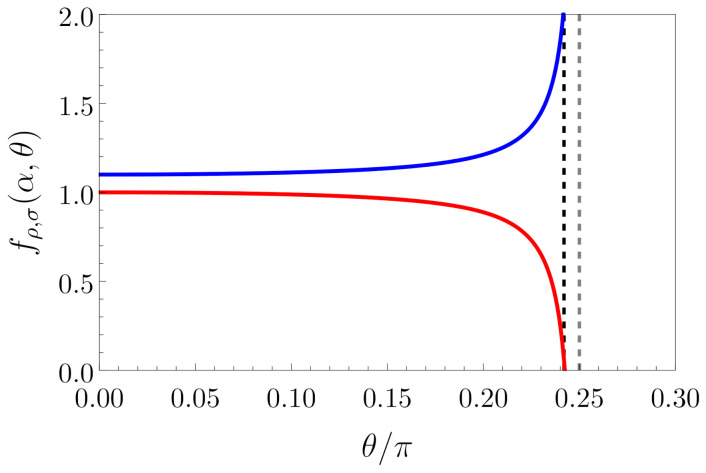
Plot of $f_{\rho }\left(\alpha ,\theta \right)$ (full blue curve) and of $f_{\sigma }\left(\alpha ,\theta \right)$ (full red curve) as a function of θ (in units of π) for fixed α=1.1. Vertical dashed lines are placed at $\theta =\frac{1}{2}\arccos\left(\frac{\alpha -1}{\alpha +1}\right)\approx 0.242\pi$ (“moderately interacting” regime in blue) and θ=π/4 (“strongly interacting” regime in gray) as references.

The stability condition of the model [48] imposes the constraint

(9) $$\theta \leq \frac{1}{2}\arccos\left(\frac{\alpha -1}{\alpha +1}\right)<\frac{\pi }{4},$$

which is manifestly more restrictive with respect to the “strongly interacting” case (α=1). The behaviors of $f_{\rho }$ and $f_{\sigma }$ as a function of θ and at fixed α are shown in Figure 1. In the following, we will focus on this general case, which seems more realistic in order to properly describe experimental observations.

## 3. Edge–Magnetoplasmon Scattering Matrix

**Figure 2:**
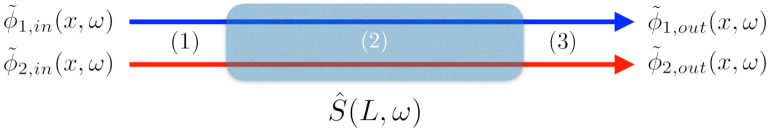
Model for a quantum Hall (QH) edge state at filling factor ν=2. According to the chirality, one can identify the incoming (injection) region (1), the interacting region (2) (shaded area of length *L*), and the outgoing (detection) region (3). In regions (1) and (3), the dynamics of the bosonic fields are well described in terms of free equations of motion (u=0). Moreover, the outgoing fields, written in the Fourier space (${\tilde{\phi }}_{i,out}\left(x,\omega \right)$, with i=1,2), are connected to the incoming ones (${\tilde{\phi }}_{i,in}\left(x,\omega \right)$, with i=1,2) through the edge–magnetoplasmon scattering matrix $\hat{S}\left(L,\omega \right)$, which encodes the information of the inter-channel interaction acting over a length *L* and at a given frequency (energy) ω.

The experiment discussed in Ref. [44] involves the injection of an electronic wave-packet with a Lorentzian profile in energy and its detection after a given propagation length along the edge. In order to describe this situation, one can proceed as in Refs. [21,40,51], where the edge channels are divided into three parts: a non-interacting injection region, an interacting propagating region, and a non-interacting region of detection (see Figure 2). Notice that this separation is not an oversimplification of the problem. Indeed, chirality guarantees that the interacting region can be made arbitrarily close both to the injection and the detection regions without loss of generality. In the following, we will discuss in detail the dynamics of the edge channels in the various regions.

- Injection region (1):
  In this region, one can assume u=0, and the Hamiltonian density can be simply written as
  (10) $$H^{\left(1\right)}\left(x\right)=\frac{v_{1}}{4\pi }{\left(\partial_{x}\phi_{1,in}\left(x\right)\right)}^{2}+\frac{v_{2}}{4\pi }{\left(\partial_{x}\phi_{2,in}\left(x\right)\right)}^{2}.$$
  The bosonic fields $\phi_{1,in}$ and $\phi_{2,in}$ propagate freely according to the equations of motion:
  (11) $$\left(\partial_{t}+v_{i}\partial_{x}\right)\phi_{i,in}\left(x,t\right)=0.$$
  By moving into a Fourier transform with respect to time, they become
  (12) $$\left(-i\omega +v_{i}\partial_{x}\right){\tilde{\phi }}_{i,in}\left(x,\omega \right)=0,$$
  with ${\tilde{\phi }}_{i,in}\left(x,\omega \right)$ field amplitudes in the frequency space defined as
  (13) $${\tilde{\phi }}_{i}\left(x,\omega \right)=\int e^{i\omega t}\phi_{i}\left(x,t\right)\;d\omega .$$
- Interacting region (2):
  In this region, the Hamiltonian density is the one in Equation (1). According to the previous discussion, the bosonic fields $\phi_{1}$ and $\phi_{2}$ are no longer eigenstates of the Hamiltonian, and the system is diagonalized in terms of a charged and a dipole mode, indicated respectively with $\phi_{\rho }$ and $\phi_{\sigma }$, with the eigenvelocities $v_{\rho }$ and $v_{\sigma }$, as discussed above. In this case, the equations of motion are
  (14) $$\left(\partial_{t}+v_{\eta }\partial_{x}\right)\phi_{\eta }\left(x,t\right)=0\;\eta =\rho ,\sigma$$
  which, expressed in a Fourier transform with respect to time, become
  (15) $$\left(-i\omega +v_{\eta }\partial_{x}\right){\tilde{\phi }}_{\eta }\left(x,\omega \right)=0.$$
  The solution of the equations of motion in this region reads
  (16) $${\tilde{\phi }}_{\eta }\left(x,\omega \right)=e^{i\frac{\omega }{v_{\eta }}x}{\tilde{\phi }}_{\eta }\left(0,\omega \right)$$
  with
  (17) $$\begin{array}{ccc} {\tilde{\phi }}_{\rho }\left(0,\omega \right) & = & \cos\theta {\tilde{\phi }}_{1,in}\left(0,\omega \right)+\sin\theta {\tilde{\phi }}_{2,in}\left(0,\omega \right)\\ {\tilde{\phi }}_{\sigma }\left(0,\omega \right) & = & -\sin\theta {\tilde{\phi }}_{1,in}\left(0,\omega \right)+\cos\theta {\tilde{\phi }}_{2,in}\left(0,\omega \right), \end{array}$$
  and the (possibly frequency-dependent) amplitudes are achieved by imposing the continuity of the fields at x=0 (boundary between regions (1) and (2)).
- Detection region (3):
  Analogously to what was discussed for region (1), also in this case, inter-channel interaction is negligible and the equations of motion are written as in Equation (11) $\left(H^{\left(1\right)}=H^{\left(3\right)}\right)$. Here, imposing the continuity of the fields at x=L (boundary between regions (2) and (3)), we observe that the outgoing field amplitudes are related to the incoming ones through the relations
  (18) $$\begin{array}{ccc} {\tilde{\phi }}_{1,out}\left(L,\omega \right) & = & \cos\theta {\tilde{\phi }}_{\rho }\left(L,\omega \right)-\sin\theta {\tilde{\phi }}_{\sigma }\left(L,\omega \right)\\ {\tilde{\phi }}_{2,out}\left(L,\omega \right) & = & \sin\theta {\tilde{\phi }}_{\rho }\left(L,\omega \right)+\cos\theta {\tilde{\phi }}_{\sigma }\left(L,\omega \right). \end{array}$$

### 3.1. Dissipative Effects

Experimental observations [39,43,44] suggest a relevant role played by energy dissipation towards additional degrees of freedom in the transport along QH edge channels. The simplest way to include this effect is by adding a real frequency-dependent energy loss rate $\gamma \left(\omega \right)$ (assumed here to be equal for both channels for the sake of simplicity) at the level of the equations of motion in the interacting region (see Equation (15)). According to this, they become

(19) $$\left[-i\omega +\gamma \left(\omega \right)+v_{\eta }\partial_{x}\right]{\tilde{\phi }}_{\eta }\left(x,\omega \right)=0.$$

In the following, we will focus on three possible behaviors for γ(ω): a non-dissipative case γ=0, a linear dissipation case $\gamma \left(\omega \right)=\gamma_{1}\omega$ ($\gamma_{1}$ real adimensional parameter) [48], and a dissipation quadratic in the energy $\gamma \left(\omega \right)=\gamma_{2}\omega^{2}$ ($\gamma_{2}$ real parameter with the dimension of a time) [43]. Notice that this additional dissipation parameter is phenomenologically accounted for by adding an imaginary term to the edge–magnetoplasmon velocities [53].

Due to this additional contribution, the solution of the equations of motion in Equation (16) acquires a frequency-dependent damping

(20) $${\tilde{\phi }}_{\eta }\left(x,\omega \right)=e^{i\left[\omega +i\gamma (\omega )\right]\frac{x}{v_{\eta }}}{\tilde{\phi }}_{\eta }\left(0,\omega \right).$$

### 3.2. General Form of the Scattering Matrix

According to the previous considerations and proceeding as in Ref. [51], we obtain the edge–magnetoplasmon scattering matrix connecting the incoming (injected) and the outgoing (detected) bosonic fields, namely:

(21) $$\left(\begin{array}{c} {\tilde{\phi }}_{1,out}\left(L,\omega \right)\\ {\tilde{\phi }}_{2,out}\left(L,\omega \right) \end{array}\right)=\hat{S}\left(L,\omega \right)\left(\begin{array}{c} {\tilde{\phi }}_{1,in}\left(0,\omega \right)\\ {\tilde{\phi }}_{2,in}\left(0,\omega \right) \end{array}\right),$$

(22) $$\hat{S}\left(L,\omega \right)=\left(\begin{array}{cc} \cos^{2}\theta e^{i\left[\omega +i\gamma (\omega )\right]\tau_{\rho }}+\sin^{2}\theta e^{i\left[\omega +i\gamma (\omega )\right]\tau_{\sigma }} & \sin\theta \cos\theta \left(e^{i\left[\omega +i\gamma (\omega )\right]\tau_{\rho }}-e^{i\left[\omega +i\gamma (\omega )\right]\tau_{\sigma }}\right)\\ \sin\theta \cos\theta \left(e^{i\left[\omega +i\gamma (\omega )\right]\tau_{\rho }}-e^{i\left[\omega +i\gamma (\omega )\right]\tau_{\sigma }}\right) & \sin^{2}\theta e^{i\left[\omega +i\gamma (\omega )\right]\tau_{\rho }}+\cos^{2}\theta e^{i\left[\omega +i\gamma (\omega )\right]\tau_{\sigma }} \end{array}\right).$$

In the above equation, we have introduced the short-hand notation $\tau_{\rho ,\sigma }=L/v_{\rho ,\sigma }$ for the times of flight associated with the propagation velocity of the charge and dipolar eigenmodes along the interacting region.

In the following, we will focus only on the top left entry of the scattering matrix in Equation (22), which represents the amplitude probability for the edge–magnetoplasmon to be transmitted along the first channel (assumed as the injection/detection channel), namely:

(23) $$\begin{array}{ccc} t\left(\omega \right) & = & \cos^{2}\theta e^{i\left[\omega +i\gamma (\omega )\right]\tau_{\rho }}+\sin^{2}\theta e^{i\left[\omega +i\gamma (\omega )\right]\tau_{\sigma }} \end{array}$$

(24) $$\begin{array}{ccc} & = & p_{\rho }\left(\theta \right)e^{i\left[\omega +i\gamma (\omega )\right]\tau_{\rho }}+p_{\sigma }\left(\theta \right)e^{i\left[\omega +i\gamma (\omega )\right]\tau_{\sigma }}. \end{array}$$

## 4. Elastic Scattering Amplitude

As discussed in Ref. [54], assuming a very peaked (ideally δ-like) injected wave-packet in energy, the relative height of this peak as a function of the energy is given, at zero temperature, by

(25) $$V\left(\varepsilon \right)=\frac{|Z\left(\varepsilon \right){|}^{2}}{|Z\left(0\right){|}^{2}}$$

with

(26) $$Z\left(\varepsilon \right)=\int_{-\infty }^{+\infty }d\tau e^{i\varepsilon \tau }\exp\left\{\int_{0}^{+\infty }\frac{d\omega }{\omega }\left[t\left(\omega \right)e^{-i\omega \tau }-1\right]e^{-\omega /\omega_{c}}\right\},$$

which is the elastic scattering amplitude (see Appendix A for more details of the calculation). Here, we introduced a converging factor $\omega_{c}$ corresponding to the greatest energy scale in the systems, and it will be sent to $\omega_{c}\to +\infty$ at the end of the calculation [55]. Notice that this picture can also be used to describe more realistic wave-packets in the energy domain as long as their width (energy dispersion) is not too large with respect to the average energy injection, a condition that is typically fulfilled in experiments [11,14,44].

In the following, we will consider the behavior of V as a function of the energy for the various possible dissipations.

### 4.1. Non-Dissipative Case

In absence of energy losses towards external degrees of freedom, the edge–magnetoplasmon transmission amplitude is

(27) $$t_{nd}\left(\omega \right)=p_{\rho }e^{i\omega \tau_{\rho }}+p_{\sigma }e^{i\omega \tau_{\sigma }}.$$

This leads, in the time domain, to

(28) $$\begin{array}{ccc} Z_{nd}\left(t\right) & = & \exp\left\{p_{\rho }\int_{0}^{+\infty }\frac{d\omega }{\omega }\left[e^{-i\omega (t-\tau_{\rho })}-1\right]e^{-\omega /\omega_{c}}\right\}\exp\left\{p_{\sigma }\int_{0}^{+\infty }\frac{d\omega }{\omega }\left[e^{-i\omega (t-\tau_{\sigma })}-1\right]e^{-\omega /\omega_{c}}\right\}\\ & = & \frac{-i}{\omega_{c}}\frac{1}{{\left(t-\tau_{\rho }-\frac{i}{\omega_{c}}\right)}^{p_{\rho }}{\left(t-\tau_{\sigma }-\frac{i}{\omega_{c}}\right)}^{p_{\sigma }}}. \end{array}$$

Its Fourier transform reads

(29) $$\begin{array}{ccc} Z_{nd}\left(\varepsilon \right) & = & \frac{-i}{\omega_{c}}\int_{-\infty }^{+\infty }dt\frac{e^{i\varepsilon t}}{{\left(t-\tau_{\rho }-\frac{i}{\omega_{c}}\right)}^{p_{\rho }}{\left(t-\tau_{\sigma }-\frac{i}{\omega_{c}}\right)}^{p_{\sigma }}}\\ & = & \frac{2\pi }{\omega_{c}}e^{i\frac{\varepsilon }{\varepsilon_{0}f_{\rho }}}{}_{1}F_{1}\left[p_{\rho },1;-i\frac{\varepsilon }{\varepsilon_{0}}\left(\frac{1}{f_{\sigma }}-\frac{1}{f_{\rho }}\right)\right]\Theta \left(\varepsilon \right) \end{array}$$

with

(30) $$\varepsilon_{0}=\frac{v}{L},$$

in which Θ(...) the Heaviside Theta function and where ${}_{1}F_{1}$ indicates the Kummer confluent hypergeometric function.

In this case, the relative height of the wave-packet evolves as

(31) $$V_{nd}\left(\varepsilon \right)=|{}_{1}F_{1}\left[p_{\rho },1;-i\frac{\varepsilon }{\varepsilon_{0}}\left(\frac{1}{f_{\sigma }}-\frac{1}{f_{\rho }}\right)\right]{|}^{2}\Theta \left(\varepsilon \right).$$

In the strongly interacting limit (α=1 and, consequently, θ=π/4), due the peculiar functional identities between hypergeometric and the zeroth-order Bessel function $J_{0}$, the above expression reduces to [54]

(32) $$Z_{nd,strong}\left(\varepsilon \right)=\frac{2\pi }{\omega_{c}}e^{i\frac{\varepsilon }{2\varepsilon_{0}}\left(\frac{1}{f_{\rho }}+\frac{1}{f_{\sigma }}\right)}J_{0}\left[\frac{\varepsilon }{2\varepsilon_{0}}\left(\frac{1}{f_{\sigma }}-\frac{1}{f_{\rho }}\right)\right]\Theta \left(\varepsilon \right),$$

with the visibility

(33) $$V_{nd,strong}\left(\varepsilon \right)=J_{0}^{2}\left[\frac{\varepsilon }{2\varepsilon_{0}}\left(\frac{1}{f_{\sigma }}-\frac{1}{f_{\rho }}\right)\right]\Theta \left(\varepsilon \right).$$

### 4.2. Linear Dissipation

The analytic expressions in this case can be obtained from the non-dissipative case by taking into account the substitution

(34) $$\omega \to \omega +i\gamma_{1}\omega$$

at the level of the first integral. This leads to

(35) $$Z_{l}\left(\varepsilon \right)=\frac{2\pi }{\omega_{c}}e^{i\frac{\varepsilon }{\varepsilon_{0}f_{\rho }}}e^{-\frac{\gamma_{1}}{f_{\rho }}\frac{\varepsilon }{\varepsilon_{0}}}{}_{1}F_{1}\left[p_{\rho },1;-\gamma_{1}\frac{\varepsilon }{\varepsilon_{0}}\left(\frac{1}{f_{\sigma }}-\frac{1}{f_{\rho }}\right)+i\frac{\varepsilon }{\varepsilon_{0}}\left(\frac{1}{f_{\sigma }}-\frac{1}{f_{\rho }}\right)\right]\Theta \left(\varepsilon \right)$$

and

(36) $$V_{l}\left(\varepsilon \right)=e^{-2\frac{\gamma_{1}}{f_{\rho }}\frac{\varepsilon }{\varepsilon_{0}}}|{}_{1}F_{1}\left[p_{\rho },1;-\gamma_{1}\frac{\varepsilon }{\varepsilon_{0}}\left(\frac{1}{f_{\sigma }}-\frac{1}{f_{\rho }}\right)+i\frac{\varepsilon }{\varepsilon_{0}}\left(\frac{1}{f_{\sigma }}-\frac{1}{f_{\rho }}\right)\right]{|}^{2}\Theta \left(\varepsilon \right).$$

### 4.3. Quadratic Dissipation

In this case, the elastic scattering amplitude can be written, in the time domain, as

(37) $$\begin{array}{cc} Z_{q}\left(t\right)\;=\; & \exp\left\{W_{\rho }\left(t\right)\right\}\exp\left\{W_{\sigma }\left(t\right)\right\}\\ =\; & \exp\left\{p_{\rho }\int_{0}^{+\infty }\frac{d\omega }{\omega }\left[e^{-i\omega (t-\tau_{\rho }-i\gamma_{2}\omega \tau_{\rho })}-1\right]e^{-\omega /\omega_{c}}\right\}\\ & \exp\left\{p_{\sigma }\int_{0}^{+\infty }\frac{d\omega }{\omega }\left[e^{-i\omega (t-\tau_{\sigma }-i\gamma_{2}\omega \tau_{\sigma })}-1\right]e^{-\omega /\omega_{c}}\right\}. \end{array}$$

This first integration can be done analytically, and the exponents $W_{\rho ,\sigma }\left(t\right)$ take the following form:

(38) $$\begin{array}{cc} W_{\rho ,\sigma }\left(t\right)=2p_{\rho ,\sigma }\{ & \gamma -\log\left(\gamma_{2}\tau_{\rho ,\sigma }\omega_{c}^{2}\right)+i\pi \;\mathrm{Erf}\left[\frac{i+\left(\tau_{\rho ,\sigma }-t\right)\omega_{c}}{2\sqrt{\gamma_{2}\omega_{c}}}\right]+\\ & -\frac{{\left(i+\left(\tau_{\rho ,\sigma }-t\right)\omega_{c}\right)}^{2}}{2\gamma_{2}\tau_{\rho ,\sigma }\omega_{c}^{2}}\;{}_{2}F_{2}\left[1,1;\frac{3}{2},2;-\frac{{\left(i+\left(\tau_{\rho ,\sigma }-t\right)\omega_{c}\right)}^{2}}{4\gamma_{2}\tau_{\rho ,\sigma }\omega_{c}^{2}}\right]\}, \end{array}$$

where γ≈0.577 is Euler’s constant and Erf is the error function. Unfortunately, it is not possible to obtain an analytical solution for the Fourier transform $Z_{q}\left(\varepsilon \right)$, and a numerical integration is needed.

## 5. Comparison with Experiments

**Figure 3:**
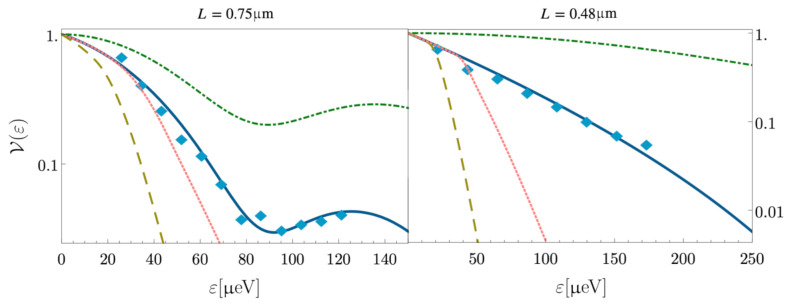
Relative peak height as a function of the injection energy (measured in μeV) for two samples of different lengths: L=0.75μm (**left panel**) and L=0.48μm (**right panel**). The non-dissipative case (green dash-dotted curve); the linear dissipative case (blue full curve) with $\gamma_{1}=0.13$ for the left panel and $\gamma_{1}=0.43$ for the right panel; quadratic dissipation with $\gamma_{2}\varepsilon_{0}=0.03$ for both panels (red dotted curve) and with $\gamma_{2}\varepsilon_{0}=0.13$ for the left panel and $\gamma_{2}\varepsilon_{0}=0.23$ for the right one (bronze dashed curve). Other parameters are: α=2.1, θ=0.17π (**left panel**) and α=1.6, θ=0.16π (**right panel**). Light-blue diamonds indicate the experimental data taken from Ref. [44].

The results obtained in the previous section are shown in Figure 3, where the relative peak height V(ε) is plotted versus the injection energy ε for two different cases that are compatible with experiments: a sample with length L=0.75μm (left panel) and one with L=0.48μm (right panel). In both panels, the parameters for the three different dissipative regimes are fixed in order to compare the theoretical expressions with the experimental data (light-blue diamonds). In the absence of dissipation along the channels (dash-dotted green line), the curve stays above the experimental data due to the absence of exponential overall decay. The observed behavior is better explained through a linear dissipation model (blue full line). The quadratic dissipation cases considered strongly deviate from the experimental situation because the decay of the relative peak height is more pronounced than the linear one. The discrepancy with the experimental data is more evident for strong dissipation (brown dashed curve) than with weak dissipation (red dotted line). According to these observations, the linear dissipation model can be considered the best candidate for describing the experimental data, at least in this case of relatively short propagation lengths (L<1 μm).

It is worth remarking that different experiments [43] that consider a regime of longer propagation lengths (L>3 μm) require one to assume a quadratic dissipation to properly reconcile theory and experiments.

## 6. Conclusions

In this paper, we have investigated the evolution of the relative peak height of electronic wave-packets well resolved in energy and ballistically propagating along QH edge channels at ν=2 as a function of the injection energy. As long as the wave-packet is narrow enough—namely, when its width is smaller with respect to the injection energy—this behavior is well described by the elastic scattering probability of the electronic excitations. In order to be close to experimental observations, we considered a wave-packet crossing an interacting region of variable length where the two edges are capacitively coupled. We assumed a short-range interaction and phenomenologically included a dissipative contribution in the model, taking into account the energy dissipation towards external degrees of freedom. According to what has been discussed in the literature, together with the conventional non-dissipative case, we considered a dissipation that is both linear and quadratic in the energy. In particular, we observed that the comparison with the experimental results discussed in Ref. [44] allows us to rule out the non-dissipative case as well as a quadratic dependence of the dissipation as a function of the injection energy, and indicates a linear energy loss rate as the more probable candidate for describing the behavior of the wave-packet for these set-ups at short enough lengths (L<1 μm). This seems to contradict what was discussed in Ref. [43], where a quadratic dissipation was indicated as the dominant contribution in the regime of long propagation length (L>3 μm). This discrepancy can be interpreted in two ways: (i) a strong sample dependence of the energy dissipation rate or (ii) more involved dissipation mechanisms, leading to different energy dependences at longer propagation lengths [46].

The present analysis has the aim of shedding new light on the behavior of electronic wave-packets propagating along ballistic mesoscopic channels and will help both theorists and experimentalists to identify new strategies for mitigating detrimental relaxation and dissipation effects.

## Data Availability

The data and analysis used in this work are available from the corresponding author upon reasonable request.

## Abbreviations

The following abbreviations are used in this manuscript:

QH	Quantum Hall
EQO	Electron Quantum Optics

## Appendix A. Calculation of the Elastic Scattering Amplitude Z

We start by considering an electron injected into channel 1 in such a way that

(A1) $$|\mathrm{in}\rangle =\int_{-\infty }^{+\infty }dy\varphi \left(y\right)\Psi^{†}{\left(y\right)|F\rangle }_{1}⊗{|F\rangle }_{2},$$

where ${|F\rangle }_{i}$ (i=1,2) is the Fermi sea associated with the *i*-th channel, $\Psi^{†}$ is the electronic creation operator, and φ(y) is its wave-packet.

In the following, we will focus on an energy-resolved wave-packet with

(A2) $$\varphi \left(y\right)=\frac{e^{i\varepsilon y}}{\sqrt{T}},$$

where the normalization T represents the longest time scale in the system and ε represents the energy.

According to the hydrodynamic approach discussed in the main text, one can write the fermionic operator acting on the Fermi sea as a coherent state of edge–magnetoplasmons (up to a Klein factor that plays no role in what follows) [47]. This leads to

(A3) $$|\mathrm{in}\rangle =\int_{-\infty }^{+\infty }dy\frac{e^{i\varepsilon y}}{\sqrt{T}}\left(\underset{\omega >0}{⨂}{|-\lambda_{\omega }\left(y\right)\rangle }_{1}\right)⊗\left(\underset{\omega >0}{⨂}{|0_{\omega }\rangle }_{2}\right),$$

where

(A4) $$\lambda_{\omega }\left(y\right)=-\frac{1}{\sqrt{\omega }}e^{i\omega y}$$

and $0_{\omega }$ is the edge–magnetoplasmon vacuum.

The analogous expression

(A5) $$|\mathrm{out}\rangle =\int_{-\infty }^{+\infty }dy^{\prime }\frac{e^{i\varepsilon y^{\prime }}}{\sqrt{T}}\left(\underset{\omega >0}{⨂}{|-\lambda_{\omega }\left(y^{\prime }\right)\rangle }_{1}\right)⊗\left(\underset{\omega >0}{⨂}{|0_{\omega }\rangle }_{2}\right)$$

holds for the state in the outgoing region.

Expressing the incoming edge–magnetoplasmons in terms of the outgoing ones requires one to take into account the entries of the matrix $\hat{S}$ in Equation (22) in such a way that

(A6) $$|\mathrm{in}\rangle \to {|\mathrm{in}\rangle }^{\prime }=\int_{-\infty }^{+\infty }dy\frac{e^{i\varepsilon y}}{\sqrt{T}}\left(\underset{\omega >0}{⨂}{|-S_{11}\left(\omega \right)\lambda_{\omega }\left(y\right)\rangle }_{1}\right)⊗\left(\underset{\omega >0}{⨂}{|-S_{12}\left(\omega \right)\lambda_{\omega }\left(y\right)\rangle }_{2}\right).$$

The elastic scattering amplitude is then given by

(A7) $$Z\left(\varepsilon \right)={\langle \mathrm{out}|\mathrm{in}\rangle }^{\prime }$$

where, taking into account the general relation for coherent states

(A8) $$\underset{\omega >0}{⨂}\langle \alpha_{\omega }|\beta_{\omega }\rangle =e^{-\frac{1}{2}\int_{0}^{+\infty }{\left|\alpha_{\omega }-\beta_{\omega }\right|}^{2}d\omega }e^{i\int_{0}^{+\infty }ℑ\left(\alpha_{\omega }^{*}\beta_{\omega }\right)d\omega },$$

with ℑ(…) representing the imaginary part, leads (in the limit T→+∞) to

(A9) $$Z\left(\varepsilon \right)=\int_{-\infty }^{+\infty }d\tau e^{i\varepsilon \tau }\exp\left\{\int_{0}^{+\infty }\frac{d\omega }{\omega }\left[t\left(\omega \right)e^{-i\omega \tau }-1\right]\right\},$$

which is the expression considered in the main text.
